# Different types of bisphenols alter ovarian steroidogenesis: Special attention to BPA

**DOI:** 10.1016/j.heliyon.2023.e16848

**Published:** 2023-06-01

**Authors:** Hamed Shoorei, Mohammad Seify, Seyedeh Fahimeh Talebi, Jamal Majidpoor, Yeganeh Koohestani Dehaghi, Majid Shokoohi

**Affiliations:** a Department of Anatomical Sciences, Faculty of Medicine, Birjand University of Medical Sciences, Birjand, Iran; b Clinical Research Development Unit of Tabriz Valiasr Hospital, Tabriz University of Medical Sciences, Tabriz, Iran; c Research and Clinical Center for Infertility, Shahid Sadoughi University of Medical Sciences, Yazd, Iran; d Student Research Committee, Birjand University of Medical Sciences, Birjand, Iran; e Department of Pharmacology, Birjand University of Medical Sciences, Birjand, Iran; f Department of Anatomy, Faculty of Medicine, Infectious Disease Research Center, Gonabad University of Medical Sciences, Gonabad, Iran

**Keywords:** Bisphenol A, Bisphenol S, Bisphenol B, Bisphenol F, Bisphenol AF, Ovary, Steroidogenesis, Hormones, Genes

## Abstract

Endocrine disruptors such as bisphenol A (BPA) and some of its analogues, including BPS, BPAF, and BPE, are used extensively in the manufacture of plastics. These synthetic chemicals could seriously alter the functionality of the female reproductive system. Although the number of studies conducted on other types of bisphenols is smaller than the number of studies on BPA, the purpose of this review study was to evaluate the effects of bisphenol compounds, particularly BPA, on hormone production and on genes involved in ovarian steroidogenesis in both in vitro (human and animal cell lines) and in vivo (animal models) studies.

The current data show that exposure to bisphenol compounds has adverse effects on ovarian steroidogenesis. For example, BPA, BPS, and BPAF can alter the normal function of the hypothalamic-pituitary-gonadal (HPG) axis by targeting kisspeptin neurons involved in steroid feedback signals to gonadotropin-releasing hormone (GnRH) cells, resulting in abnormal production of LH and FSH. Exposure to BPA, BPS, BPF, and BPB had adverse effects on the release of some hormones, namely 17-β-estradiol (E2), progesterone (P4), and testosterone (T). BPA, BPE, BPS, BPF, and BPAF are also capable of negatively altering the transcription of a number of genes involved in ovarian steroidogenesis, such as the steroidogenic acute regulatory protein (StAR, involved in the transfer of cholesterol from the outer to the inner mitochondrial membrane, where the steroidogenesis process begins), cytochrome P450 family 17 subfamily A member 1 (Cyp17a1, which is involved in the biosynthesis of androgens such as testosterone), 3 beta-hydroxysteroid dehydrogenase enzyme (3β-HSD, involved in the biosynthesis of P4), and cytochrome P450 family 19 subfamily A member 1 (Cyp19a1, involved in the biosynthesis of E2). Exposure to BPA, BPB, BPF, and BPS at prenatal or prepubertal stages could decrease the number of antral follicles by activating apoptosis and autophagy pathways, resulting in decreased production of E2 and P4 by granulosa cells (GCs) and theca cells (TCs), respectively. BPA and BPS impair ovarian steroidogenesis by reducing the function of some important cell receptors such as estrogens (ERs, including ERα and ERβ), progesterone (PgR), the orphan estrogen receptor gamma (ERRγ), the androgen receptor (AR), the G protein-coupled estrogen receptor (GPER), the FSHR (follicle-stimulating hormone receptor), and the LHCGR (luteinizing hormone/choriogonadotropin receptor). In animal models, the effects of bisphenol compounds depend on the type of animals, their age, and the duration and dose of bisphenols, while in cell line studies the duration and doses of bisphenols are the matter.

## 1 Introduction

Bisphenol A (BPA) and many other bisphenol types such as bisphenol B (BPB), bisphenol Z (BPZ), bisphenol F (BPF), bisphenol S (BPS), bisphenol AP (BPAP), tetra bromo bisphenol A (TBBPA), as well as bisphenol AF (BPAF) are synthetic substances, commonly used for more than 50 years in polycarbonate plastics such as medical devices, toys, food containers, plastic bottles, dental sealants, etc. [1,2]. Although there are a number of different types of bisphenols, as mentioned above, the effects of some of them, such as BPA, BPS, BPS, BPAF, and BPF, on ovarian folliculogenesis have been studied by scientists.

BPA is a well-known endocrine-disrupting chemical (EDC) with a global consumption of 7.7 million tonnes in 2015 and about 10.6 million tonnes in 2022 [3], and some of its analogues such as mentioned earlier have shown various harmful properties such as reproductive toxicity, neurotoxicity, and genotoxicity [4]. BPA has been shown to cause epigenetic changes by altering DNA methylation [5]. Several studies showed that BPA could disturb the expression of some genes involved in steroidogenesis by altering DNA methylation levels of Cyp17a1 and Cyp11a1 and histone methylation [6,7]. In addition, BPA has both estrogenic and antiandrogenic properties. It could be a ligand for many receptors, such as the nonclassical membrane-bound G-coupled protein receptor [8], the orphan estrogen-related receptor gamma (ERRγ) [9], and the two classical oestrogen receptors-α and β (ERα [ESR1] and ERβ [ESR2]) [10], and it can also act as an antagonist when it binds to the androgen receptor (AR) [11].

It has been reported that even low doses of BPA can disrupt hormone balance [12] and interact with steroidogenic enzymes [13]. BPA has also been shown to disrupt ovarian folliculogenesis in animals [14], alter the number of ovarian follicles [15], increase the number of atretic follicles [16], and alter the estrous cycle [17]. In granulosa cells (GCs) from humans or mice treated with BPA, an increase in the Bax/Bcl-2 ratio and G2-M arrest [18] and promotion of autophagy via mediation of the AMPK/mTOR/ULK1 pathway [19] resulted in DNA damage and cell death [18,20].

Several in vivo studies showed that BPA involved apoptosis in ovaries (Caspase-3, Fas/FasL, and Bax), [[21], [22]], induced autophagic vesicle formation in ovarian GCs [23], and altered genes involved in steroidogenesis [24]. In addition, early exposure to BPA has been reported to increase the number of atretic follicles, while decreasing ovarian size and antral follicle number [[25], [26]] .

A number of previous studies have shown that BPS and BPF affect the male/female ratio [27], blood androgen and estrogen levels [28], and the plasma levels of estradiol (E2), progesterone (P4), testosterone (T), luteinizing hormone (LH), and follicle-stimulating hormone (FSH) [[29], [30]]. The non-conjugated form of BPS has been shown to be more detectable in blood than BPA due to its higher bioavailability [31]. An animal study showed that the administration of BPA, BPB, BPF, and BPS could increase the number of atretic and cystic follicles and decrease the number of antral and corpus luteum follicles [32], resulted in a change in the production of steroid hormones.

Although the adverse effects of BPA, BPE, BPS, BPF, and BPAF on ovarian function have been demonstrated in several studies, this review article aims to examine the relationship between ovarian steroidogenesis and various types of bisphenols, especially BPA, because BPA is much more in the focus of scientists' interest in research.

## 2 The hypothalamic-pituitary-ovarian (HPO) axis

**Fig. 1:**
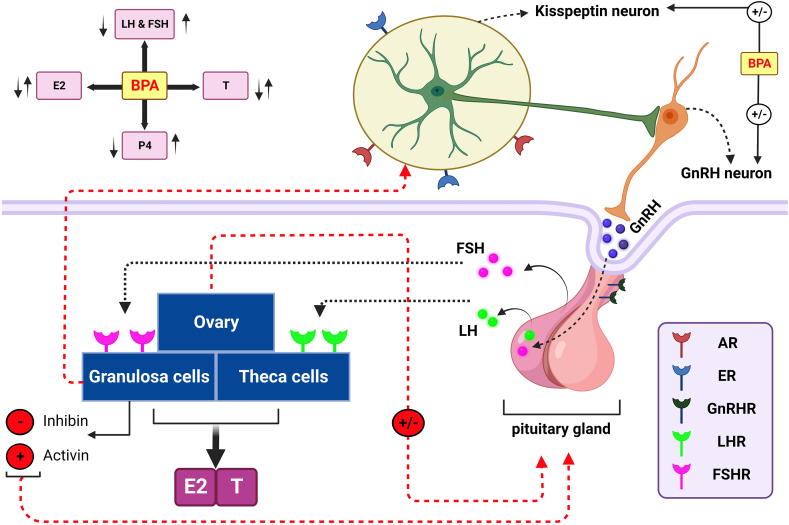
Bisphenol compounds alter the levels of hormones released by the ovaries and the anterior pole of the pituitary gland. This figure shows how bisphenol compounds, especially BPA, alter the function of the HPO axis. In the hypothalamus, the kisspeptin neuron could affect ovarian activities through its action on GnRH neurons. GnRH modulates the secretion of FSH and LH from the anterior pituitary by binding to its receptor (GnRHR). FSH and LH act by binding to their receptors (FSHR and LHR) on ovarian granulosa and theca cells to regulate oogenesis and steroidogenesis by inducing the production of E2, T, and P4. Bisphenol compounds may alter levels of the hormones estrogen, progesterone, and testosterone produced by the ovaries by interfering with the function of the HPO axis.

Although normal reproductive function in women depends on normal ovarian activity, the secretion of gonadotropin-releasing hormone (GnRH) from the hypothalamus and the subsequent release of gonadotropins (LH and FSH) from the pituitary gland are exerted as primary controls. Therefore, the HPO axis plays an important role in female reproduction. The released GnRH, after entering the pituitary portal circulation, binds to the GnRH receptors (GnRHR) located on the anterior pituitary to stimulate the secretion of LH and FSH from the gonadotropins into the circulation. Subsequently, by binding to their receptors (LHR and FSHR) located on the thecal and granulosa cells, respectively, these two secreted hormones stimulate the production of sex steroids such as P2, E2, and T in females to support folliculogenesis. On the other hand, both positive and negative feedback effects of sex steroids might be involved in GnRH secretion. Although the androgen receptor (AR) and/or estrogen receptor α (ERα) are not expressed in GnRH neurons in the hypothalamus, upstream GnRH neurons, such as hypothalamic kisspeptin neurons (KISS), which express both AR and ERα, are involved in steroid feedback signals to GnRH cells (Fig. 1).

### 2.1 BPA alters the function of the HPO axis

EDCs may have antagonistic or agonistic effects through their action on androgen and estrogen receptors. Evans et al. reported that BPA exposure at later developmental time points alters the secretion of gonadotropins in prepubertal female lambs [33]. Brannick et al. reported that BPA exposure (in utero) at a low dose can increase gonadotropin cell numbers, because estrogenic compounds increase the proliferation of the pituitary cells [34]. The levels of lhβ and fshβ decreased when mice were treated with 50 μg/kg, whereas their levels increased when mice were treated with 0.5 μg/kg [34]. BPA exposure during the neonatal period resulted in a decrease in Kiss1 levels in rats [[35], [36], [37]] and overexpression of Kiss1 levels in fish [38], CD-1 mice [39], and rats [[40], [41]].

During the above period, exposed BPA altered ESR1, ESR2, and GnRH (Gnrh) levels in some animals, including rats and sheep [[35], [38], [42], [43]]. A low dose of BPA (20 μg/kg) to sexually mature mice during the proestrus cycle was also able to increase plasma levels of some hormones (E2, FSH, and LH), mRNA expression of Gnrh and Kiss1, and protein concentration of kisspeptin in the anteroventral periventricular nucleus (AVPV) [42].

Xi et al. reported that BPA exposure resulted in the upregulation of FSH levels in female pups at the hypothalamic-pituitary level [39]. BPA could activate the HPO axis by enhancing AVPV kisspeptin[44]. Therefore, the function of the HPO axis could be mediated by the kisspeptin signaling pathway. Oral administration of BPA (50 mg/kg) from postnatal day 19 to day 68 increased mRNA expression of GnRHR, GRP54 (a G protein-coupled receptor and a target for the KISS1 gene), and GnRH genes and decreased the density of ER in female adolescent rats, resulting in overproduction of E2, FSH, and LH [45] (Fig. 1).

Lower expression of ER may be due to excessive production of estrogen hormones. It has been reported that excessive expression of GPR54 leads to the overproduction of estrogen and LH through the activation of the gonadotropic axis [46]. In addition, BPA can decrease progesterone receptor (PgR) expression and could also inhibit the ability of progesterone to counteract E2 action; therefore, this could be considered a reason why BPA exposure could increase E2 levels [47].

### 2.2 Other types of bisphenols alter the function of the HPO axis

Several studies showed that BPF, BPS, BPB, BPAF, and BPA (100 μg/L) can increase mRNA expression of fshβ, lhβ, gnrh3, kiss1, and kiss2 in the neuroendocrine system of zebrafish embryos, leading to an increase in the levels of E2, LH, and FSH [[48], [49]]. Long-term exposure to BPF did not alter the expression levels of kiss2 or kisspeptin receptor 2 (kiss2r) in zebrafish’ brain, but increased the expression of some genes (kiss1, kiss1r, gnrh3, lhβ, and fshβ) and the levels of LH and FSH [50]. Zebrafish embryos receiving BPF (0.25 and 0.5 μM) showed a remarkable reduction in the development of the GnRH neuronal system, especially in GnRH3 neurons in the preoptic area (POA) and terminal nerve (TN) involved in reproductive behavior [51]. Zebrafish embryos exposed to a broad spectrum of BPF were able to alter gene transcription in the hypothalamic-pituitary-thyroid (HPT) axis and stimulate the expression of estrogen receptor markers [[52], [53]].

Therefore, the mechanism of action of bisphenols might be due to their effects on the Kiss/GnRH signaling pathway (Fig. 1). The contrasting results could also be due to age or species of the animals, the dose of the exposed BPA or the other bisphenol types (low or high), and/or the duration of exposure. However, we strongly suggest that the use of kisspeptin and Gpr54 knock-out mice may be very useful to study the effects of BPA or other bisphenol types on the hypothalamus and pituitary gland.

## 3 Ovarian steroidogenesis

**Fig. 2:**
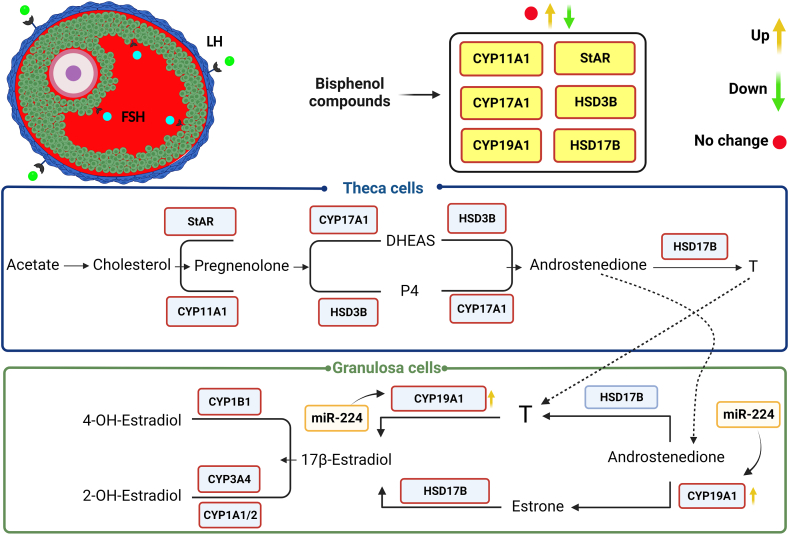
Bisphenol compounds, especially BPA, alter the expression of genes involved in ovarian steroidogenesis. In follicles (generally preantral, antral, and preovulatory follicles), cooperative interaction between granulosa and theca cells leads to ovarian steroidogenesis. Within the theca cells and in response to LH, cholesterol is converted to androgens via a complicated signaling pathway, while another complicated signaling pathway is required for the conversion of androgens from the theca cells to estrogens. Bisphenol compounds, by suppressing/activating the expression of the above enzymes involved in steroidogenesis, such as StAR, Cyp19a1, and Hsd3b, could ultimately affect the production of E2, P4, and T.

Steroidogenesis (Fig. 2) is a process in which important sex steroids such as E2, P4, and T are formed. The first step of steroidogenesis is initiated when steroidogenic acute regulatory protein (StAR) transports cholesterol from the outer mitochondrial membrane (OMM) to the inner mitochondrial membrane (IMM), the site where cholesterol is converted to pregnenolone by binding to the cholesterol side-chain cleavage enzyme (P450scc [CYP11A1]) [54]. Pregnenolone is converted to a weak androgen, dehydroepiandrosterone (DHEA), via an intermediate steroid, 17-hydroxypregnenolone (17OH-Preg), involving the enzyme 17α-hydroxylase-17,20-desmolase (CYP45017α; CYP17A1), or to progesterone via the enzyme 3β-hydroxysteroid dehydrogenase (HSD3β) [55]. Subsequently, androstenedione can be formed from DHEA (via HSD3β) and progesterone (via CYP17A1). Within the theca cells, androstenedione is converted to testosterone by the enzyme 17β-hydroxysteroid dehydrogenase (HSD17B) [55]. Some of the androstenedione in theca cells diffuses into the granulosa cells where the aromatase enzyme (CYP450arom; CYP19A1) converts androstenedione to estrone or HSD17B converts androstenedione to testosterone [55]. Estrone and testosterone are then converted to E2 via the HSD17B and CYP19A1 enzymes, respectively. Interestingly, two other forms of E2, 2-hydroxyestradiol (2- OH -estradiol) and 4-hydroxyestradiol (4- OH -estradiol) can cause cell damage and cell death via inactivation of E2 [56,57].

On the other hand, FSH could increase the expression of the CYP450arom enzyme by binding to its receptor, FSHR, located on granulosa cells and by activating cAMP-dependent protein kinase A [58]. However, several other factors such as peroxisome proliferator-activated receptor gamma (PPAR-γ), steroidogenic factor 1 (SF -1), and insulin-like growth factors (IGFs) might be involved in CYP450arom activation [59,60]. In addition, LH could increase the activity of enzymes involved in T and androstenedione synthesis by binding to its receptor LHR located on theca cells [61].

Because bisphenol compounds have widely varying effects on the production of hormones and genes involved in steroidogenesis, the goal of this review is to examine these effects in both in vivo and in vitro studies.

## 4 BPA alters the production of sex hormones (LH and FSH), steroid hormones (E2, P4, and T), and genes involved in steroidogenesis

### 4.1 Human studies

It has been reported that in women with polycystic ovary syndrome (PCOS), urinary BPA concentration is significantly increased [62,63]. Yang et al. indicated that exposure to BPA can trigger a PCOS-like syndrome [26]. Jafar & Rasheed found that serum BPA, T, and cholesterol levels in PCOS patients were 45.3 pg/mL, 1.53 ng/mL, and 207 mg/mL, respectively [64]. Miao et al. showed that in female workers exposed to BPA in China, urinary BPA concentration (22.2 μg/g) decreased compared to the unexposed group, serum levels of P4 and FSH did not change significantly, LH levels (1.27 mIU/mL) decreased, and E2 levels (3.87 pg/mL) increased compared to the unexposed group [65].

In infertile women, although a positive association between steroid hormones and the presence of BPA is still controversial, a positive correlation between BPA and the interaction of free and total testosterone and dehydroepiandrosterone sulfate (DHEA-S) has been demonstrated [62,66,67]. Therefore, in women exposed to BPA, serum levels of FSH, LH, T, E2, and P4 could change dramatically depending on the duration of exposure and BPA concentration.

### 4.2 In vivo studies (animal models)

Gámez and colleagues reported that early exposure to a low dose of BPA (3 μg/kg) increased serum levels of LH and E2 but observed no change in FSH levels [25]. Liang et al. showed that maternal exposure to BPA (250 mg/kg/day) resulted in a significant change in hormone levels such as E2 and T in the offspring of rats [68]. Exposure of BPA (50, 500, and 2500 mg/kg) to the mother of mice resulted in a dose-dependent decrease in T and FSH levels and an increase in E2 production in the adult offspring (at 8 weeks of age) [69]. It has been suggested that lower T levels may result from suppressed GnRH, leading to inhibition of gonadotropin secretion (Fig. 1), [70]. In addition, Lite et al. found in 12-week-old female rat offspring that serum levels of E2 and expression of miR-224 increased in their pregnant BPA-exposed mothers, while serum FSH levels decreased [71]. It has been reported that miR-224 could trigger TGF-β signaling pathway involved in folliculogenesis via interaction with SMAD family member 4 (SMAD4) protein [72,73]. and could also promote the release of estradiol from GCs by regulating the expression of Cyp19a [[73], [74], [75]], (Fig. 2). Therefore, an increase in E2 levels in the offspring of rats could be due to the effects of BPA on the expression of microRNAs such as miR-224, leading to a change in the expression of genes involved in steroidogenesis and subsequently to a lower/higher production of estrogens.

Santamaría et al. reported that in female rats born to mothers treated with BPA (0.5 and 50 μg/kg), serum levels of E2 and mRNA levels of Cyp11, Cyp17, LHCGR, and Cyp19 did not change at postnatal day 90, whereas transcription of FSHR and Hsd3b and production of P4 increased [76]. Therefore, the upregulation of FHSR seems to be a compensatory mechanism for the increase in E2 production to reach the standard level, while the high P4 levels may be due to the overexpression of Hsd3b.

Nguyen et al. found that in animals prenatally exposed to BPA, the mRNA expression level of Cyp17a1 (a key enzyme in the biosynthesis of androgens) decreased in postnatal day 1 females, whereas the protein level of steroid-5β-reductase/aldo-keto-reductase 1D1 (AKR1D1) increased in postnatal day 21 females [77] may encode enzymes involved in the catalysis of steroid hormones, with an exception for estrogens [78]. Therefore, in postnatal animals, the balance of steroid hormones might be altered due to a change in the transcription of Akr1d1. Also, Silva et al. reported that both low and high doses of BPA (10 or 50 μg/kg) during gestation (postnatal day 21) and lactation (postnatal day 180) can induce lifelong changes by affecting sex steroid hormones, including increased T and P levels and also decreased E2 levels [79]. However, female rats had regular estrous cycles, but ovarian mass increased in the BPA10-treated group only at PN180 [79]. Therefore, any hormonal changes in early life could affect ovarian development, which is reflected in morphological changes in adulthood. Wei et al. found that exposure to BPA (0–40 mg/kg/day, gestational day 0.5–17.5) during pregnancy decreased serum levels of FSH, E2, and P4 in F1 females at postnatal day 21 and 56, while serum levels of LH increased and decreased at postnatal day 21 and 56, respectively [7]. Moreover, in FVB mice (F0) exposed to different doses of BPA (0.5, 20, or 50 μg/kg/day, as in utero), serum levels of E2 increased in the F1 generation (at postnatal day 4) but not in the other generations, F2 and F3 [21]; also, mRNA expression of StAR (increased in the F2 generation), Cyp17a1 (increased in all generations), and Hsd17b1 (increased in the F1 and F2 generations) in a dose-dependent manner [21]. Mahalingam et al. showed that utero BPA exposure (20 and 50 μg/kg) could decrease E2 (in the F1 generation of mice) and T (in the F2 generation of mice) and did not affect P4 in both F1 and F2 generations [15]. They also reported that in the F1/F2 generation of mice (at 3 months of age), BPA at any dose did not affect the mRNA levels of StAR, Cyp11a1, Cyp19a1, and Hsd17b1, whereas in the F2 generation of mice (at 12 months of age), expression of StAR, Cyp11a1, and Hsd3b1 decreased, whereas transcription of Cyp19a1 and Hsd17b1 increased in a dose-dependent manner [15]. The decreased E2 levels in BPA-treated animal models could be due to the reduction in the number of preantral follicles or to a change in the activity of factors/genes involved in steroidogenesis and/or the conversion of cholesterol to E2 [15]. It has been suggested that increased E2 levels in the F1 generation may be due to a reduction in the degradation of germ cell nests, whereas BPA in the F2 and F3 generations may inhibit the degradation of germ cell nests. Therefore, it appears that the effects of BPA on E2 levels are not transgenerational.

Thilagavathi et al. showed that long-term treatment with BPA (10, 50, and 100 μg/kg, 3 months) resulted in a decrease in serum levels of E2 and P4, as well as mRNA expression of StAR and protein levels of Cyp11a1, respectively, while serum levels of FSH and LH increased in SD rats treated with 10 μg BPA [80]. Also, oral administration of BPA (daily, with a wide dose range of 0.05–1 kg/BW) for 13 months to female Wistar rats increased serum T and androstenedione concentrations for all BPA concentrations [81]. Increased plasma androstenedione concentrations may be due to 1) inhibition of T catabolism [82], 2) inhibition of androgen receptor activity such that synthesis of androstenedione and T increased due to decreased bound androgen levels [83].

Several studies in fish reported that BPA could lead to an increase in plasma E2 levels and a decrease in T levels [[84], [85], [86]]. In the ovary of fish exposed to BPA (15 μg/L, 1 week), transcription of Cyp19a increased [87], while treatment with 50 μg/L BPA decreased the expression of Cyp19a in female minnows [88]. Paitz & Bowden found that the ability of redband mucosa (*Trachemys scripta*) embryos to convert estrone to estrone sulfate decreased when BPA was administered [89]. Zhang et al. showed that two weeks of treatment with BPA (15 μg/L) significantly increased serum levels of E2 and transcription of Hsd11b2 (involved in the conversion of active cortisol to inactive cortisone) and also suppressed the expression of StAR (by increasing the methylation of StAR through altering the expression of DNA methyltransferases [DNMTs]) in female rare minnows Gobiocypris rarus (G. rarus) [90]. Rhee et al. also found an increase in mRNA transcription of StAR in Kryptolebias marmoratus exposed to BPA (600 μg/L, for 4 days) [91]. Therefore, the difference in the expression of StAR could be due to the age of the animals or the concentration of bisphenols. Additionally, Faheem et al. reported that administration of BPA at various doses (10–1000 μg/L, for 2 weeks) resulted in an increase in plasma levels of E2 (100 and 1000 μg/L BPA), a decrease in plasma levels of T (1000 μg/L BPA), and a significant increase in plasma levels of FSH (10 μg/L BPA) and LH (1000 μg/L BPA) in female Catla fish [92]. It is also presented that BPA could dose-dependently increase the mRNA transcription of FSHR, StAR, and Cyp19a (involved in the conversion of C19 androgens to C18 estrogens). Therefore, the increased E2 levels and decreased T production could be due to the upregulation of two genes, including FSHR (involved in estradiol production) and Cyp19a transcription (involved in the conversion of T to estrogens).

In G. rarus exposed to short-term treatment with BPA (15 μg/L, 21 days), the levels of E2 and T increased, mRNA expression of StAR, Cyp11a1, and Cyp17a1 decreased, and the level of Cyp19a1a increased; mid-term treatment (42 days) increased the production of E2; and finally, with long-term treatment with BPA (63 days), both E2 and T levels decreased, mRNA expression of StAR, Cyp11a1, and Cyp17a1 increased, and Cyp19a1a levels decreased [6]. The exact mechanism of how short-, mid-, and long-term treatment with BPA might affect the production of E2 and T in G. rarus is not clear. Finally, BPA might have differential effects on the production of hormones and genes involved in steroidogenesis in animals, and these effects might depend on the generation/age of the animals and the dose of BPA.

### 4.3 In vitro studies (human and animal cell lines)

BPA at a dose of 10 μM was able to decrease the P4 level in human ovarian GCs (KGN), increased the mRNA expression of StAR, and did not alter the expression levels of Hsd3b2 (an important gene responsible for progesterone biosynthesis) and Cyp11a1 [93]. Shi et al. reported that a broad spectrum of BPA (0–500 μM, for 6 h) was able to interfere with steroid hormone synthesis in KGN cells, while all of the above doses were unable to markedly alter the expression of FHSR and StAR [94]. On the one hand, they have also found that in KGN cells exposed to BPA, mRNA expression of p450 oxidoreductase (i.e. POR (involved in the synthesis of androstenedione) increased, protein levels of G-proteins (GS) decreased, production of adenylate cyclase (AC) increased, expression levels of Hsd17b2 and Hsd17b3 (involved in the synthesis of T) and a number of genes such as ferredoxin and ferredoxin reductase (involved in the synthesis of P4) were downregulated [94]. The GS/AC pathway is responsible for the conversion of androstenedione to T via targeting 17βHSD. Therefore, the main reason that BPA does not lead to a significant change in T secretion from KGN cells exposed to BPA could be due to overexpression of POR and/or a decrease in the efficiency of conversion of androstenedione to testosterone. Reduction in E2 production could also result from the decomposition of E2 to 2/4- OH estradiol by Cyp1b1 or Cyp1a1 [95]; J [94]. or due to insufficient production of testosterone by POR to produce E2 [[94], [95]]. or due to suppression of Hsd17b1, resulting in restriction of the pathway that converts estrone to E2 [94].

Wu et al. showed that E2 levels increased in GCs from pigs exposed to BPA (10 μM, for 24–48 h), possibly due to the inductive effects of BPA on granulosa cell proliferation [96]. Exposure of GCs isolated from immature rats to BPA (100 μM, for 48 h) resulted in decreased secretion of P4, decreased mRNA transcription of the ATP-binding cassette subfamily A member 1 gene (ABCA1), and also increased mRNA expression of StAR, Cyp11a1, and Hsd3b1, as well as protein levels of sterol regulatory element-binding protein-1 (SREBP-1) [97]. In this context, ABCA1 may be involved in the storage of intracellular cholesterol required for steroidogenesis as a reverse cholesterol transporter. It has been reported that both protein and mRNA expression of ABCA1 could be affected by BPA, and gene polymorphism of ABCA1 could also lead to severe PCOS by affecting steroid [93,97,98]. Moreover, any disturbance in cholesterol homeostasis or imbalance between cholesterol uptake and export in GCs may lead to lower secretion of P4 [97]. Moreover, sterol-regulating element-binding proteins are responsible for controlling the transcription of various types of genes involved in the process of de novo cholesterol synthesis [99]. Therefore, any attenuation of the production of P4 from GCs could be due to the disruptive effects of BPA on cholesterol homeostasis by targeting StAR/SREBP-1 and decreasing the expression of ABCA1 [99]. Mlynarcikova & Scsukova found that P4 production and mRNA expression of StAR, Cyp11a1, and Hsd3b decreased when porcine ovarian GCs were treated with BPA (100 μM, for 3 days) [100]. It was suggested that the lower production of P4 may be due to the lower viability or metabolic activity of GCs [100].

## 5 Other types of bisphenols alter the production of sex hormones (LH and FSH), steroid hormones (E2, P4, and T), and genes involved in steroidogenesis

### 5.1 In vivo studies (animal models)

In CD-1 mice prenatally exposed to BPA and two other analogues, BPE and BPS (0.5, 20, and 50 μg/kg/day) from gestational day 11 to birth, serum levels of T (in the BPE- and BPS -treated groups) increased, whereas E2 levels did not change in the next generation at 3, 6, and 9 months of age [101]; furthermore, in the next generation of mice (at 3, 6, and 9 months of age), mRNA expression of StAR, Cyp11a1, Cyp17a1, Cyp19a1, Hsd17b1, and Hsd3b1 changed in response to different doses of BPA, BPE, and BPS [101]. Ahsan and colleagues showed in female rats neonatally exposed to a wide range of BPS and BPA (0–50 mg/kg, from postnatal day 1–10 that plasma concentrations of T and E2 increased, whereas levels of P4, LH, and FSH decreased at postnatal day 75 [102]. Ijaz et al. investigated the effects of BPA and some of its alternatives such as BPS, BPF, and BPB (5–50 mg/kg, for 28 days) as separate substances on the serum concentrations of E2, P4, and T in female rats after weaning [32]. In this context, all the mentioned types of bisphenols increased the serum concentrations of T, while they decreased the concentrations of E2 and P4 in a dose-dependent manner [32].

In zebrafish exposed to BHPF, the expression of Cyp17a1 and Cyp19a1 was significantly downregulated, while the mRNA expression of Cyp11a1 increased [103].

### 5.2 In vitro studies (human and animal cell lines)

Amar et al. showed that the production of P4 and E2 decreased in a dose-dependent manner when human GCs were treated with BPS [104]. In addition, a wide dose range of BPS from 10 nM to 50 μM failed to alter mRNA expression levels of StAR, Cyp17a1, and Hsd3b, but the transcription of Cyp11a1 changed [104]. It has been reported that although mitogen-activated protein kinase3/1 (MAPK3/1) expression on ovarian GCs is critical for steroidogenesis [105], BPA and BPS can alter MAPK3/1 expression. Indeed, in sheep GCs, a difference was found between the effects of BPS and BPA on MAPK3/1 signaling, such that treatment with BPA was able to inhibit MAPK3/1 phosphorylation, whereas no such effect was observed for BPS [106]. Moreover, Majkic et al. found that MAPK3/1 phosphorylation increased in human GCs exposed to 100 μM BPA for 30 min [107], whereas no significant change in MAPK3/1 phosphorylation was observed in another study in which human GCs were exposed to 10 μM BPS [104]. Therefore, the effects of BPA on the MAPK3/1 signaling pathway might be more deleterious than BPS.

Mlynarcikova & Scsukova found that in GCs of pigs treated with different types of bisphenol compounds such as BPA, BPAF, BPS, and BPF (10-9 - 10-4 M, for 24–72 h), BPA and BPAF (10-4 M) were able to decrease P4 levels, while the production of E2 decreased in a dose-dependent manner in both BPS and BPAF-treated groups [108]. In addition, all of the aforementioned bisphenol compounds failed to alter the mRNA expression of StAR, Forkhead Box O1 (FOXO1), Cyp11a1, Cyp19a1, and Hsd3b [108]. FOXO1 has been reported to act as a key regulator of GC functions by modulating some genes involved in steroidogenesis [109,110]. Therefore, the fact that the expression of the aforementioned genes involved in steroidogenesis does not change could be due to the lack of change in the expression of the FOXO1 gene [108]. Berni et al. reported that in GCs isolated from adult porcine ovaries and treated with BPS (1 and 10 μM, for 48 h), the production of E2 decreased, whereas P4 levels did not change significantly [111]. Decreased expression of Cyp19a1 was also observed in cumulus cells from pigs treated with BPS (30 μM, for 48 h) [112]. Campen and colleagues added a wide range of BPS to the culture media of bovine GCs and TCs and found that the production of androstenedione and P4 was not affected by BPS, whereas treatment with 100 μM BPS increased E2 levels E2 [113]. It has been suggested that BPS can be converted to glucuronide- and sulfate-conjugated forms, and the estrogenic activity of BPS in these forms is significantly lower than in the unconjugated [114]. Increased E2 levels at high doses of BPS could therefore be due to the aforementioned fact.

Long-term exposure of preantral follicles to BPS (0.1 and 10 μM, for 15 days) decreased E2 production in a dose-dependent manner, did not alter P4 secretion and follicular survival, and had no effect on mRNA expression of Cyp19a1 and Hsd3b1 [115]. Therefore, the lack of effect of BPS on progesterone hormone secretion could be due to a lack of change in Hsd3b1 expression.

Finally, it appears that BPS may decrease the production of E2 and P4 if the dose is too high to inhibit the proliferation of GCs [111]. Moreover, any change in StAR gene expression seems to depend on the duration of exposure, the concentration of EDCs, and the type of cells. Therefore, BPA and its analogues such as BPE and BPA have adverse effects on StAR expression in a dose-dependent manner.

## 6 Effects of bisphenol compounds, especially BPA and BPS, on estrogen, progestogen, and androgen receptors (ER, PgR, and AR)

A wide range of molecular action has been reported for BPA, such as the ability to bind to ERα and ERRγ membranes, acting as an estrogen receptor antagonist and agonist (both ERα and ERβ), and acting as an antagonist for the androgen receptor [116]. On the other hand, estrogen receptor agonist activity (via ERα/B) has been suggested for BPS in several studies [117,118]. In growing follicles, ERα is expressed in theca/granulosa cells as well as in stromal/interstitial cells, whereas ERβ and AR are mainly expressed in GCs and the highest levels are observed in GCs of antral follicles [76]. Both ERα and ERβ might mediate the stimulatory effect of estrogens, especially E2, on granulosa cell proliferation [119]. Moreover, ERRγ is involved in estrogen signaling and cellular energy metabolism [120].

On the other hand, BPA, BPE and BPB have been suggested to have the highest affinity for ERRγ [121]. and also the affinity of bisphenols for ERα and ERβ is categorized as BPFA > BPB > BPA > BPF > BPS [122]. Therefore, any alteration in the expression and function of all the above receptors impairs follicular maturation and leads to abnormal production of ovarian hormones.

### 6.1 In vivo studies (animal models)

During pregnancy, exposure to different doses of BPA could increase mRNA expression of ERα and PgR, while BPA at the doses of 5 and 10 mg/kg could increase mRNA expression of ERβ in the ovary of F1 mice at postnatal day 21 [7]. However, in the ovaries of F1 mice at postnatal day 56, the mRNA levels of ERα, ERβ, and PgR decreased [7]. BPA could form a complex by binding to estrogen receptors, namely BPA-ERs [123]. This complex mimics the estradiol- ER complex and, by binding to the estrogen response element (ERE), alters transcription factors involved in the activation of weak estrogen action [123]. Therefore, BPA as a synthetic estrogen could vaporize the weak estrogen during mouse development and increase the expression of ERα and PgR in the ovaries before sexual maturation.

Zhang et al. showed that exposure to BPA could suppress the transcription of ESR1 (after 7 days) and upregulate the transcription of ESR2b (after 14 days), whereas BPA could not affect the expression of ESR2a during the two-time points mentioned above [90]. Therefore, any change in the expression of steroidogenic genes after exposure to BPA could be attributed to the altered transcripts of ERs. In zebrafish treated with a substitute of bisphenol A, fluorene-9-bisphenol (BHPF, 300 mg/L), for 1 month, steroid hormone biosynthesis was disrupted when the expression of ESR1 and ESR2α was downregulated [103]. BPA exposure in F0 mice failed to alter ESR1 and AR in the F1 generation (postnatal day 21), but the levels of ESR1 and AR increased in the F2 generation, and the expression of ESR1 also decreased in the F3 generation [21]. Since AR and ESR1 are significantly involved in the process of folliculogenesis, their upregulated expression in the F2 generation could be due to the decrease in the number of primordial follicles in the same generation, while the lower expression of ESR1 in the F3 generation could demonstrate the fact that BPA could affect the receptors differently in each generation.

Clarisa et al. found that treatment with low doses of BPA (0.5 and 50 μg/kg) during gestation and lactation failed to alter protein expression of ERα and ERβ in the female offspring of rats at postnatal day 90, while the expression of AR in primary follicles of mice, whose mothers were treated with 0.5 μg/kg. Also, the number of several follicles, including primary, preantral, and antral, in mice whose mothers were treated with 50 μg/kg was reduced [76]. Low doses of antiandrogens can increase the ovulation response, while high doses can decrease the number of ovulations [124]. Therefore, a decrease in protein expression of AR in preantral and antral follicles could result from an increase in ovulation rate, a slight decrease in androgen action, and/or a decrease in the transition of the follicle from primordial to primary [76].

Hill et al. reported that BPS exposure during the developmental period failed to alter mRNA expression of ESR1 and ESR2 in the ovaries of CD-1 mice [125]. Developmental exposure to BPS (200 μg/kg, daily, from gestational day 8 to postnatal day 19) in CD-1 mice significantly increased ovarian follicle development (particularly the number of secondary follicles) increased epidermal growth factor 1 (EGF-R) and insulin growth factor 1 (IGF-1) mRNA expression, but did not alter ERα and ERβ mRNA expression [125]. IGF-1 and EGF-R may be regulated by estrogen during folliculogenesis [126,127] and the increased number of mature follicles after treatment with BPS may result from increased expression of IGF-1 and EGF-R genes [125].

### 6.2 In vitro studies (human and animal cell lines)

Long-term exposure of preantral follicles to BPS (0.1 and 10 μM, 15 days) did not alter mRNA expression of ESR1 and ESR2 [115]. Administration of BPS (10 nM–50 μM) was able to dose-dependently alter mRNA expression of ERRγ and G protein-coupled estrogen receptor (GPER) in human GCs, whereas mRNA expression of PgR, ESR1, and ESR2 did not change [104]. Although the exact role of GPER in ovarian tissues remains to be elucidated, GPER is thought to be responsible for E2-mediated stimulation of primordial follicle formation and non-genomic estrogen-related signaling [128]. Therefore, a decrease in E2 as well as P4 levels in human GCs when exposed to BPS may be related to abnormal expression of ERRγ and GPER [104].

## 7 Conclusion

BPA and its analogues, as endocrine-disrupting chemicals, have controversial effects on the production of sex and steroid hormones and on genes involved in steroidogenesis in ovarian tissue. However, the exact mechanism of their action is so complicated that it has not yet been fully elucidated.

Exposure to BPA, BPS, BPF, BPAF, and BPB may affect gene expression involved in steroidogenesis (such as Cyp19a1, Hsd3B, and StAR), apoptosis (such as caspase-3 and p53), cell cycle (such as cyclin-E1 and cyclin-D2). Exposure to different types of bisphenols could alter the number of follicles at different stages. Thus, they can increase the number of atretic follicles, but decrease the number of other follicles, such as antral, through the induction of apoptotic genes such as Bax and caspase-3. By decreasing the number of antral follicles, the production of E2 and P4 may decrease, and through a negative feedback loop, the production of FSH and LH is increased. Also, they can increase E2, P4, and T concentration and decrease LH and FSH production by triggering a number of genes such as fshβ, lhβ, gnrh3, kiss1, ERα/β, etc. Therefore, the effects of bisphenols are quite different compared to each other.

Also, any disruption of the normal function of the hypothalamic-pituitary axis by kisspeptin and gnrh genes, leading to alteration in the secretion of LH and FSH. In addition, several in vitro studies have reported that BPA is able to change the secretion of E2 and P4 from granulosa and theca cells, respectively. Interestingly, the type of animals, their age, and the duration and dose of bisphenol compounds have many different effects on HPO axis, genes involved in steroidogenesis, and hormone productions by ovaries, whereas, in vitro studies, the duration and doses of BPA and/or its analogues and the type of cell line are important.

## Author contribution statement

All authors listed have significantly contributed to the development and the writing of this article.

## Data availability statement

Data will be made available on request.

## Additional information

No additional information is available for this paper.

## Declaration of competing interest

None.
